# Fault-Tolerant Control of Autonomous Underwater Vehicle Actuators Based on Takagi and Sugeno Fuzzy and Pseudo-Inverse Quadratic Programming under Constraints

**DOI:** 10.3390/s24103029

**Published:** 2024-05-10

**Authors:** Zimu Zhang, Yunkai Wu, Yang Zhou, Dahai Hu

**Affiliations:** 1 School of Electrical and Information Engineering, Jiangsu University, Zhenjiang 212013, China; 3210507016@stmail.ujs.edu.cn; 2 College of Automation, Jiangsu University of Science and Technology, Zhenjiang 212100, China; hu1350118711@163.com; 3 School of Computer Science and Engineering, Jiangsu University of Science and Technology, Zhenjiang 212100, China; jsjxy_zy@just.edu.cn

**Keywords:** AUV actuators, fault-tolerant control, T-S fuzzy, quadratic programming, control constraints

## Abstract

Autonomous Underwater Vehicles (AUVs) play a significant role in ocean-related research fields as tools for human exploration and the development of marine resources. However, the uncertainty of the underwater environment and the complexity of underwater motion pose significant challenges to the fault-tolerant control of AUV actuators. This paper presents a fault-tolerant control strategy for AUV actuators based onTakagi and Sugeno (T-S) fuzzy logic and pseudo-inverse quadratic programming under control constraints, aimed at addressing potential actuator faults. Firstly, considering the steady-state performance and dynamic performance of the control system, a T-S fuzzy controller is designed. Next, based on the redundant configuration of the actuators, the propulsion system is normalized, and the fault-tolerant control of AUV actuators is achieved using the pseudo-inverse method under thrust allocation. When control is constrained, a quadratic programming approach is used to compensate for the input control quantity. Finally, the effectiveness of the fuzzy control and fault-tolerant control allocation methods studied in this paper is validated through mathematical simulation. The experimental results indicate that in various fault scenarios, the pseudo-inverse combined with a nonlinear quadratic programming algorithm can compensate for the missing control inputs due to control constraints, ensuring the normal thrust of AUV actuators and achieving the expected fault-tolerant effect.

## 1. Introduction

Autonomous Underwater Vehicles (AUVs) play a crucial role in the exploration and utilization of marine resources and space. As a new tool for exploring the ocean, AUVs significantly enhance the efficiency of underwater detection and operations. They play an extensive role in various areas, such as marine resource exploration, underwater engineering operations, scientific research and surveys. As AUVs need to work long-term in the ocean, the harsh conditions and complex and ever-changing environment may cause various faults in the actuators of their motion control systems. Therefore, fault-tolerant control technology has become an urgent need to enhance the safety and reliability of AUVs.

Fault tolerance refers to a system’s ability to detect and diagnose its own faults in the event of component faults, such as actuators, sensors or controlled objects, and to initiate appropriate corrective actions based on the diagnosis results. This enables the system to continue functioning as intended while maintaining performance consistency with a fault-free scenario. Fault-tolerant control can be categorized into passive fault-tolerant control and active fault-tolerant control based on its characteristics. Passive fault-tolerant control typically employs a fixed controller structure and parameters, utilizing robust control techniques to render the system insensitive to specific faults [1]. Passive fault-tolerant control is characterized by the ability of the original controller to mitigate the effects of a component failure, ensuring that the system can continue to carry out its tasks with reduced performance. In Ref. [2], a fault-tolerant controller is designed for the depth control of an AUV. The controller utilizes periodic output feedback gains and a multi-model approach. In the event of thruster failure, the multi-model system adjusts by using a gain matrix with all off-diagonal terms set to zero. Ref. [3] proposed a finite-time extended state observer (FTESO)-based scheme for AUV fault-tolerant control problems with multiple-thrusters by estimating the uncertainties caused from faults, modeling uncertainty and system disturbances.

Active fault-tolerant control relies on real-time fault diagnosis information to modify the control inputs of the system to maintain stability. This approach can be classified into two categories based on how the control inputs are adjusted: fault accommodation and control reconfiguration [4]. Control reconfiguration involves the real-time reconstruction of the control system based on fault diagnosis results using specific algorithms. In Ref. [5], genetic algorithms are incorporated into the reconstruction of fault-tolerant control laws to address reliability issues in AUVs. The proposed method utilizes constrained genetic algorithms to reconstruct fault-tolerant control laws for AUVs. By providing relevant fault weight matrices under different fault scenarios, the genetic algorithm is used to search for the optimal solution of the control matrix, facilitating the reconstruction of a propulsion system control matrix. In addressing the actuator fault-tolerant control issue, control reconstruction is accomplished by leveraging the inherent redundant actuators of the robot, thereby enabling the fault-tolerant control of the AUV, as discussed in Refs. [6,7]. A reconfigurable control allocation scheme was proposed in Ref. [8] for possible types of fault in X-rudder UUV, which allows the UUV to operate normally even in cases of partial rudder failure, and its effectiveness was verified through simulation. An observer-based fault-tolerant control scheme for a discrete-time descriptor system with signal-to-noise ratio-constrained channels was proposed in Ref. [9]. In this framework, the augmented robust filter can achieve the simultaneous estimation of system states and faults. The introduction of dynamic thresholds further enhances the performance of this diagnostic method, providing theoretical guidance for the design of fault-tolerant control for AUVs.

Fault regulation involves adjusting the controller parameters or structure based on fault diagnosis information to maintain the input–output relationship between the controller and the controlled system. Ref. [10] developed a continuous adaptive nonlinear model by adjusting the controller or model parameters. In the event of an actuator fault, neural networks and fuzzy logic are employed to modify the model parameters, enabling fault-tolerant control. Refs. [11,12] focused on thrust allocation modeling and fault-tolerant control techniques, utilizing recursive neural networks for remote-controlled AUVs. Ref. [13] established a control energy function using the duality principle, which is based on the thrust allocation model. This approach enables the implementation of a recursive neural-network-based fault-tolerant control algorithm for the thrusters. Ref. [14] proposed a thrust allocation method based on the minimum l2 norm for a specific type of 7000-m manned submersible manufactured by China Shipbuilding Industry Corporation. The correctness and effectiveness of the control allocation algorithm were verified using a hybrid allocation algorithm based on the pseudoinverse matrix and fixed-point allocation on a semi-physical simulation platform. Ref. [15] decoupled fault-tolerant control from a dynamic controller design and used nonlinear programming to minimize allocation errors and control costs as optimization objectives during the control allocation process to solve the fault-tolerant control problem. Ref. [16] proposed a hybrid algorithm based on singular-value decomposition and fixed-point allocation for fault-tolerant control allocation in AUV propulsion systems. Compared to traditional methods, the above-mentioned approach reduces computational complexity by avoiding the problem of pseudoinverse matrix calculation and can satisfy the thruster saturation constraints.

Currently, there is a scarcity of literature on passive fault-tolerant control methods. Reliable stabilization is one of the passive fault-tolerant control methods for controller faults. This concept involves employing multiple compensators in parallel to stabilize the same controlled object [17]. Ref. [18] demonstrated that the essential condition for reliable stabilization with two compensators is the strong stabilizability of the controlled object. Ref. [19] addressed the aforementioned problem by introducing a parameterized approach for designing two dynamic compensators, offering a solution to the reliable stabilization problem. Ref. [20] developed a solution for the reliable stabilization problem by utilizing multiple dynamic compensators for multivariable systems that are not strongly stabilizable. Ref. [21] investigated the integrity concern associated with open circuit faults, proposed a method to solve the static feedback gain matrix, and provided a numerical solution method for the integrity issue of the closed-loop system configuration within actuator faults. Ref. [22] proposed a state feedback law design with actuator fault integrity control, utilizing optimization-solving through numerical solutions of compatible nonlinear equation systems. However, the integrity concern was not entirely resolved. The main challenge lies in the lack of an effective constructive method for solving fault-tolerant control laws, particularly in the study of high-dimensional multivariable systems [23,24]. As a result, this area presents potential research prospects.

This paper presents a fault-tolerant control strategy for AUV actuators, utilizing T-S fuzzy control and pseudoinverse quadratic programming under control constraints. The subsequent sections of this article are structured as follows: Section 2 covers the design of the T-S fuzzy controller. Section 3 delves into the fault-tolerant control of the actuator. Section 4 focuses on the fault-tolerant control of actuators under control constraints. Experimental verification and analyses are shown in Section 5. Conclusions and our future work are presented in Section 6.

## 2. System Description

### 2.1. T-S Fuzzy Modelling

**Figure 1:**
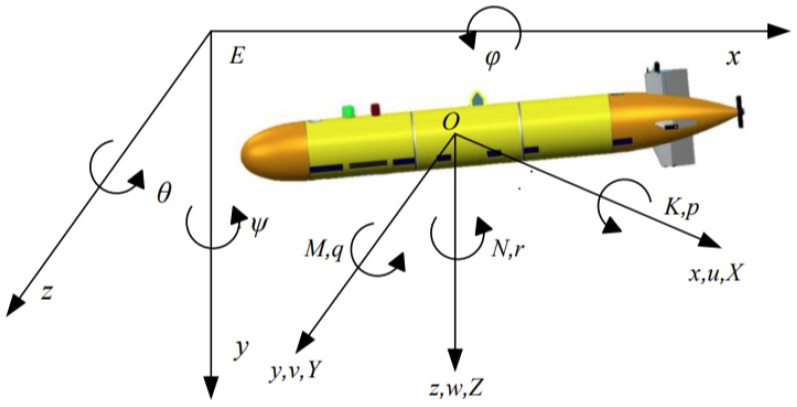
Coordinate diagram for AUV.

The underwater dynamics of an AUV are influenced by buoyancy, gravity, thrust, hydrodynamic forces, interference forces, etc. The model in both the static coordinate system and the dynamic coordinate system is illustrated as in Figure 1. Furthermore, the six-degrees-of-freedom motion equations of AUV can be found in to Ref. [25], with detailed definitions of parameters provided in Table 1.

The AUV underwater motion is a six-degrees-of-freedom spatial motion, which can be represented by linear motion along three axes and rotational motion around three axes in a dynamic coordinate system. Assume that the center of gravity of the AUV (*G*) does not coincide with the origin of the dynamic coordinate system (*O*), and its coordinates in the dynamic coordinate system are $x_{g}$, $y_{g}$, $z_{g}$. For the dynamics equation of the AUV, if only the thrust and torque of the actuators are considered, the rigid-body six-degrees-of-freedom spatial motion equation of the AUV can be represented as follows [25]:

(1) $$\left\{\begin{array}{c} X=m\left[u-vr+wq-x_{g}\left(r^{2}+q^{2}\right)+y_{g}\left(pq-r\right)+z_{g}\left(pr+q\right)\right]\\ Y=m\left[v-ur-wp-y_{g}\left(r^{2}+p^{2}\right)+y_{g}\left(qr-p\right)+x_{g}\left(pq+r\right)\right]\\ Z=m\left[w-uq+vp-z_{g}\left(p^{2}+q^{2}\right)+x_{g}\left(pr-q\right)+z_{g}\left(qr+p\right)\right]\\ K=I_{x}p+\left(I_{z}-I_{y}\right)qr+m\left[y_{g}\left(w-uq+vp\right)-z_{g}\left(v+ur-wp\right)\right]\\ M=I_{x}q+\left(I_{x}-I_{z}\right)pr+m\left[z_{g}\left(u-vr+wq\right)-x_{g}\left(w+vp-uq\right)\right]\\ N=I_{x}r+\left(I_{z}-I_{y}\right)pq+m\left[x_{g}\left(v-ur+wp\right)-y_{g}\left(u+vr-wq\right)\right] \end{array}\right.$$

where *m* is the mass of the AUV; $\rho_{A}$ is the density of the AUV; $I_{x}$, $I_{y}$ and $I_{z}$ are the moments of inertia, which can be represented as follows:

(2) $$\left\{\begin{array}{c} I_{x}=\int_{V}\left(y^{2}+z^{2}\right)\rho_{A}dV\\ I_{y}=\int_{V}\left(x^{2}+z^{2}\right)\rho_{A}dV\\ I_{z}=\int_{V}\left(x^{2}+y^{2}\right)\rho_{A}dV \end{array}\right.$$

Furthermore, for AUV actuator fault modeling, this article establishes a parameter deviation model for AUV output thrust and torque, shown as follows:

(3) $$T=(T_{n\left|n\right|}+b_{n})n\left|n\right|$$

(4) $$M=M_{\delta }\left(\delta +b_{\delta }\right)u^{2}$$

where *T* is the main thrust, $T_{n\left|n\right|}$ is the main thrust coefficient, $b_{n}$ is the parameter deviation of main thrust, *n* is the propeller speed, *M* is the thrust torque, $M_{\delta }$ is the rudder angle coefficient, δ is the rudder angle, and $b_{\delta }$ is the rudder angle deviation. The propeller and rudder are operating normally when $b_{n}=0$ and $b_{\delta }=0$. Applying the aforementioned actuator fault description to the AUV nonlinear dynamics, one can obtain the following:

(5) $$\begin{array}{c} m\left(\dot{u}-vr+wq\right)=X_{u\left|u\right|}u\left|u\right|+X_{\dot{u}}\dot{u}+X_{vr}vr\\ +\left(T_{n\left|n\right|}+b_{Ln}\right)n_{L}\left|n_{L}\right|+\left(T_{n\left|n\right|}+b_{Rn}\right)n_{R}\left|n_{R}\right| \end{array}$$

(6) $$m\left(\dot{v}+ur\right)=Y_{\dot{v}}\dot{v}+Y_{\dot{r}}\dot{r}+Y_{ur}ur+Y_{uw}uw+Y_{\delta }\left(\delta +b_{\delta }\right)u^{2}$$

(7) $$\begin{array}{cc} I_{z}\dot{r}= & N_{v\left|v\right|}v\left|v\right|+N_{r\left|r\right|}r\left|r\right|+N_{\dot{v}}\dot{v}+N_{\dot{r}}\dot{r}+N_{ur}ur+N_{uv}uv+N_{\delta }\left(\delta +b_{\delta }\right)u^{2}\\ \; & +\left(\left(T_{n\left|n\right|}+b_{Ln}\right)n_{L}\left|n_{L}\right|-\left(T_{n\left|n\right|}+b_{Rn}\right)n_{R}\left|n_{R}\right|\right)d \end{array}$$

where the distance *d* is the distance between the left and right thrusters and the longitudinal centerline of the AUV.

To address the issue of fault-tolerant control allocation for the AUV actuator, it is essential to provide a detailed description of the mathematical models involved.

The AUV considered in this paper is a small, torpedo-shaped AUV. Considering the modeling of its actuators, the following form of the nonlinear dynamic system is considered:

(8) $$\left\{\begin{array}{c} \dot{x}\left(t\right)=f\left(x\left(t\right)\right)+g\left(x\left(t\right)\right)u\left(t\right)\\ y\left(t\right)=Cx\left(t\right) \end{array}\right.$$

where $x\left(t\right)={\left[u\;\;v\;\;w\;\;q\;\;r\;\right]}^{T}$ is the state vector of the system; $u\left(t\right)$ is the control input to the system; $f\left(\cdot \right)$ is the nonlinear term of the system; $g\left(x\left(t\right)\right)$ is the control input matrix for the system; $y\left(t\right)$ is the output of the system; *C* is the known constant coefficient matrix.

We initially constructed nonlinear T-S fuzzy models for the AUV control system, based on which the state feedback-based fuzzy controllers utilizing these models were developed. The T-S fuzzy model aims to approximate nonlinear systems by employing multiple local linear models established according to the IF-THEN rules. These rules effectively transform intricate nonlinear problems into familiar linear ones [27]. Each rule corresponds to a subsystem within a nonlinear system that illustrates the dynamic characteristics within each local region. Global nonlinearity is attained through fuzzy inference, derived from local linearization.

For the control system of AUV (8), the fuzzy rules are formulated as follows:

$$Rule\;i\;:\;IF\;\theta_{1}\left(t\right)\;is\;M_{1}^{i}\;and\;\cdots \;and\;\theta_{r}\left(t\right)\;is\;M_{r}^{i};$$

Then

(9) $$\left\{\begin{array}{c} \dot{x}\left(t\right)=A_{i}x\left(t\right)+B_{i}u\left(t\right)\;i=1,2,\cdots ,r\\ y\left(t\right)=Cx\left(t\right) \end{array}\right.$$

where $M_{1}^{i},\;M_{2}^{i},\cdots ,\;M_{r}^{i}$ are fuzzy sets; $A_{i},\;B_{i},\;C_{i}$ are known real number matrices with corresponding dimensions; $\theta_{1}\left(t\right),\;\theta_{2}\left(t\right),\cdots ,\;\theta_{r}\left(t\right)$ are known prior variables, which can be a state variable, external disturbance, a time function, or a combination of these vectors.

By integrating the individual subsystems using methods such as single-point fuzzification, product inference, and weighted average defuzzification, we can derive the ultimate hybrid model of the nonlinear system, as illustrated below. The state equation of the entire fuzzy system can be expressed as follows:

(10) $$\left\{\begin{array}{c} \dot{x}\left(t\right)=\sum_{i=1}^{r}\mu_{i}\left(\theta \left(t\right)\right)\left(A_{i}x\left(t\right)+B_{i}u\left(t\right)\right)\\ y\left(t\right)=\sum_{i=1}^{r}\mu_{i}\left(\theta \left(t\right)\right)Cx\left(t\right) \end{array}\right.$$

where $\mu_{i}\left(\theta \right)=\frac{\omega_{i}\left(\theta \left(t\right)\right)}{\sum_{i=1}^{r}\omega_{i}\left(\theta \left(t\right)\right)}$ is the membership function, $\omega_{i}\left(\theta \left(t\right)\right)=\prod_{j=1}^{s}M_{j}^{i}\left(\theta_{j}\right)$. The following conditions are satisfied:

(11) $$\mu_{i}\left(\theta \left(t\right)\right)>0,\sum_{i=1}^{r}\mu_{i}\left(\theta \left(t\right)\right)=1$$

The importance of constructing a fuzzy model lies in its ability to approximate a nonlinear dynamic model as an ensemble of local linear models, thereby enhancing fuzzy precision by incorporating a larger number of fuzzy rules. However, as the number of fuzzy rules increases, the design intricacy of fuzzy controllers also increases. Therefore, system modeling must strike a balance between accuracy and complexity.

**Figure 2:**
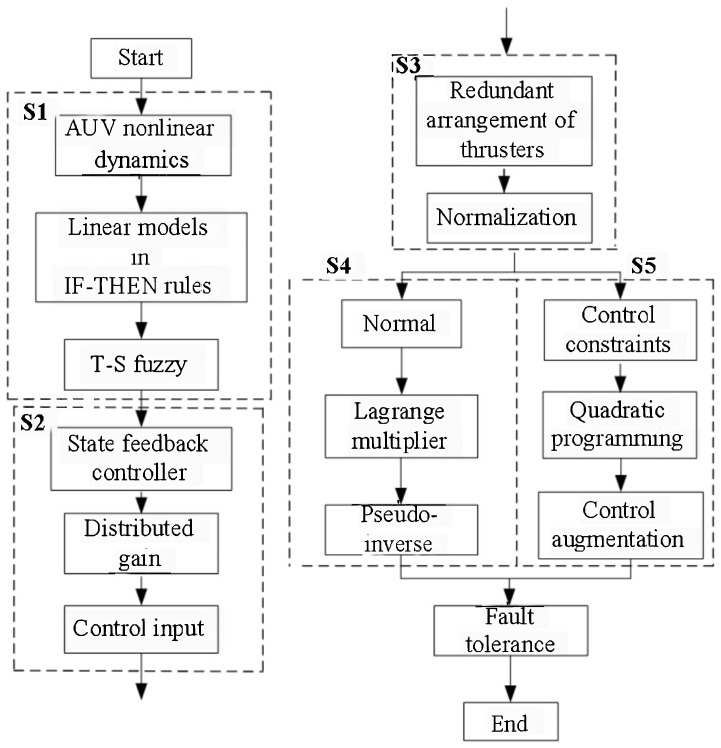
The flowchart of fault-tolerant control scheme.

In order to better elucidate the fault-tolerant control scheme proposed in this article, the flowchart shown as in Figure 2 is provided. Specific steps will be detailed in subsequent chapters.

### 2.2. The Design and Stability of Fuzzy Controllers

When designing controllers for T-S fuzzy models, the prevalent approach in the current research involves the utilization of the parallel distribution compensation method. In the controller design, the premise variables of each controller’s fuzzy rule are the same as the corresponding premise variables of the fuzzy model. Subsequently, a state feedback control law is formulated for each rule’s respective linear subsystem, followed by the derivation of the control law for the global system through fuzzification weighting.

For the T-S fuzzy system in Section 2.1, the state feedback controller is structured based on the parallel distribution compensation algorithm. The controller rules are outlined as follows:

$$Rule\;i:\;IF\;\theta_{1}\left(t\right)\;is\;M_{1}^{i}\;and\;\cdots \;and\;\theta_{r}\left(t\right)\;is\;M_{r}^{i};$$

Then

(12) $$u\left(t\right)=K_{j}x\left(t\right)\;j=1,\;2,\cdots ,\;r$$

where $K_{j}$ is the gain matrix for the compensation of the controller distribution that needs to be designed. By amalgamating the controllers of the aforementioned subsystems, the comprehensive fuzzy controller can be acquired, as depicted below:

(13) $$u\left(t\right)=\sum_{j=1}^{r}\mu_{j}\left(\theta \left(t\right)\right)K_{j}x\left(t\right)$$

Furthermore, a fuzzy model of the closed-loop system is obtained, as follows:

(14) $$\left\{\begin{array}{c} \dot{x}\left(t\right)=\sum_{i=1}^{r}\sum_{j=1}^{r}\mu_{i}\left(\theta \left(t\right)\right)\mu_{j}\left(\theta \left(t\right)\right)\left(A_{i}+B_{i}K_{j}\right)x\left(t\right)\\ y\left(t\right)=\sum_{i=1}^{r}\mu_{i}\left(\theta \left(t\right)\right)Cx\left(t\right) \end{array}\right.$$

When designing a system, the stability of the control system should be prioritized to guarantee the smooth operation of the system. To establish a robust stability criterion for the fuzzy control system (14), a controller can be designed from a theoretical standpoint.

Take the Lyapunov function as $V\left(x\left(t\right)\right)={\left[x\left(t\right)\right]}^{T}Px\left(t\right)$, where *P* is a symmetric and positive-definite matrix; then, one can obtain the following:

(15) $$\begin{array}{cc} \dot{V}\left(x\left(t\right)\right) & ={\left[\dot{x}\left(t\right)\right]}^{T}Px\left(t\right)+{\left[x\left(t\right)\right]}^{T}P\dot{x}\left(t\right)\\ \; & ={\left[\sum_{i=1}^{r}\sum_{j=1}^{r}\mu_{i}\left(\theta \left(t\right)\right)\mu_{j}\left(\theta \left(t\right)\right)\left(A_{i}+B_{i}K_{j}\right)x\left(t\right)\right]}^{T}Px\left(t\right)\\ \; & +{\left[x\left(t\right)\right]}^{T}P\left[\sum_{i=1}^{r}\sum_{j=1}^{r}\mu_{i}\left(\theta \left(t\right)\right)\mu_{j}\left(\theta \left(t\right)\right)\left(A_{i}+B_{i}K_{j}\right)x\left(t\right)\right]\\ \; & =\sum_{i=1}^{r}\sum_{j=1}^{r}\mu_{i}\left(\theta \left(t\right)\right)\mu_{j}\left(\theta \left(t\right)\right){\left[x\left(t\right)\right]}^{T}\left[{\left(A_{i}+B_{i}K_{j}\right)}^{T}P\right.\\ \; & \left.+P\left(A_{i}+B_{i}K_{j}\right)\right]x\left(t\right) \end{array}$$

For the formula (15), the Left and right side of ${\left(A_{i}+B_{i}K_{i}\right)}^{T}P+P\left(A_{i}+B_{i}K_{j}\right)$ are multiplied by $P^{-1}$ as $P^{-1}{\left(A_{i}+B_{i}K_{j}\right)}^{T}+\left(A_{i}+B_{i}K_{j}\right)P^{-1}$.

Denote $H_{j}=K_{j}P^{-1}$

(16) $$Q_{ij}={\left(A_{i}R+B_{i}H_{j}\right)}^{T}+\left(A_{i}R+B_{i}H_{j}\right)$$

The variables in the Equation (16) must satisfy the following conditions:

(17) $$\left\{\begin{array}{c} Q_{ij}<0\;\;i=1,\;2,\cdots ,\;r\\ \frac{1}{r-1}Q_{ij}+0.5\left(Q_{ij}+Q_{ji}\right)<0\;\;1\leq i\neq j\leq r \end{array}\right.$$

When the aforementioned conditions are fulfilled, $\dot{V}\left(x\left(t\right)\right)<0$, then the T-S fuzzy system exhibits asymptotic stability.

### 2.3. The Design of T-S Fuzzy Rules

The variations in the state variables v,w,q and r in the six-degrees-of-freedom motion equations of an AUV are interconnected with the linear velocity *u*. Changes in *u* will impact the velocities and angular velocities of other degrees of freedom. Additionally, there is a constraint relationship between *q* and *r* in the posture equation, as both are influenced by the control moments simultaneously. To facilitate future research, it is essential to simplify the variables needed for the fuzzy controller design. Denote $\theta_{1}=u,\;\theta_{2}=r$; choose $\theta_{1}$ and $\theta_{2}$ as the premise variables of the T-S fuzzy system. According to the actual situation, the corresponding fuzzy sets are designed as follows: $\left\{\theta_{1}=0.5,\;1\right\},\;\left\{\theta_{2}=-0.1,\;0,\;0.1\right\}$.

The commonly used membership function information includes the upper and lower bounds of the membership function, as well as boundary information. For instance, one can approximate the membership function using a ladder function or by selecting a certain number of equally spaced points and approximating the information with adjacent points. Generally, the membership function in a fuzzy model typically mirrors that of a fuzzy controller, requiring the consideration of accuracy requirements and computational complexity. In the context of the actual AUV system, we select the following membership functions for the AUV actuator fuzzy controller:

(18) $$M_{\theta_{1}=0.5}=\frac{2+\sin\left(\theta_{1}\left(t\right)\right)}{5},M_{\theta_{1}=1}=\frac{3-\sin\left(\theta_{1}\left(t\right)\right)}{5}$$

(19) $$\begin{array}{cc} M_{\theta_{2}=-0.1} & =\frac{\cos\left(\theta_{2}\left(t\right)\right)+2}{5},M_{\theta_{2}=0}=\frac{\sin\left(\theta_{2}\left(t\right)\right)+3}{5},\\ M_{\theta_{2}=0.1} & =\frac{-\sin\left(\theta_{2}\left(t\right)\right)-\cos\left(\theta_{2}\left(t\right)\right)+5}{5} \end{array}$$

For Equations (18) and (19), six working points are selected with reference to the fuzzy set $\left[0.5,-0.1\right],\left[0.5,0\right],\left[0.5,0.1\right],\left[1,-0.1\right],\left[1,0\right],\left[1,-0.1\right]$. Thus, the design of AUV fuzzy model and fuzzy controller are as follows:

Rule1: $IF\;\;\theta_{1}\;\;is\;\;about\;\;0.5\;m/s\;and\;\theta_{2}\;is\;about\;-0.1\;rad/s,then$

(20) $$\left\{\begin{array}{c} \dot{x}\left(t\right)=A_{1}x\left(t\right)+B_{1}u\left(t\right)\\ u\left(t\right)=K_{1}x\left(t\right) \end{array}\right.$$

Rule2: $IF\;\;\theta_{1}\;\;is\;\;about\;\;0.5\;m/s\;and\;\theta_{2}\;is\;about\;0\;rad/s,then$

(21) $$\left\{\begin{array}{c} \dot{x}\left(t\right)=A_{2}x\left(t\right)+B_{2}u\left(t\right)\\ u\left(t\right)=K_{2}x\left(t\right) \end{array}\right.$$

Rule3: $IF\;\;\theta_{1}\;\;is\;\;about\;\;0.5\;m/s\;and\;\theta_{2}\;is\;about\;0.1\;rad/s,then$

(22) $$\left\{\begin{array}{c} \dot{x}\left(t\right)=A_{3}x\left(t\right)+B_{3}u\left(t\right)\\ u\left(t\right)=K_{3}x\left(t\right) \end{array}\right.$$

Rule4: $IF\;\;\theta_{1}\;\;is\;\;about\;\;1\;m/s\;and\;\theta_{2}\;is\;about\;-0.1\;rad/s,then$

(23) $$\left\{\begin{array}{c} \dot{x}\left(t\right)=A_{4}x\left(t\right)+B_{4}u\left(t\right)\\ u\left(t\right)=K_{4}x\left(t\right) \end{array}\right.$$

Rule5: $IF\;\;\theta_{1}\;\;is\;\;about\;\;1\;m/s\;and\;\theta_{2}\;is\;about\;0\;rad/s,then$

(24) $$\left\{\begin{array}{c} \dot{x}\left(t\right)=A_{5}x\left(t\right)+B_{5}u\left(t\right)\\ u\left(t\right)=K_{5}x\left(t\right) \end{array}\right.$$

Rule6: $IF\;\;\theta_{1}\;\;is\;\;about\;\;1\;m/s\;and\;\theta_{2}\;is\;about\;0.1\;rad/s,then$

(25) $$\left\{\begin{array}{c} \dot{x}\left(t\right)=A_{6}x\left(t\right)+B_{6}u\left(t\right)\\ u\left(t\right)=K_{6}x\left(t\right) \end{array}\right.$$

In the above rules, the controller distribution compensation gain vector of each subsystem is as follows: $K=\left[K_{1}\;\;K_{2}\;\;K_{3}\;\;K_{4}\;\;K_{5}\;\;K_{6}\right]$. By combining the known membership functions, a complete fuzzy controller can be designed to provide control input for the AUV motions and provide the premise for the subsequent fault-tolerance control.

## 3. Fault-Tolerant Control of Auv Actuators

### 3.1. Arrangement and Normalization Strategy of the Propulsion System

**Figure 3:**
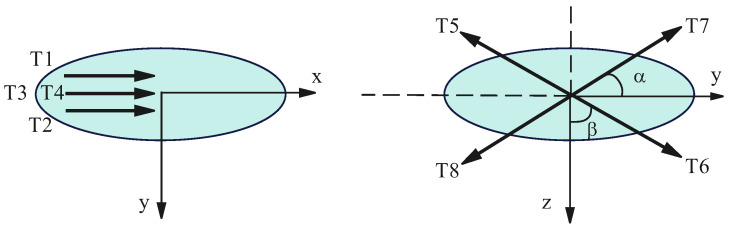
Propulsion system layout for AUV.

The devices collectively powering the AUV are known as its propulsion system, which includes propellers and rudder engines (as shown in Figure 3). The propellers consist of two horizontal thrusters and two vertical thrusters, producing thrusts T1, T2, T3, T4 (actually, T1 and T2 are the same, described as u1; T3 and T4 are the same, described as u2), located at the tail of the AUV. The rudder engines consist of vertically and horizontally distributed rudders that produce thrust moments T5, T6, T7, T8 (where T5 and T6 are denoted as u3, and T7 and T8 are represented as u4). The propulsion system of the AUV has the ability to directly control all six degrees of freedom, making it equipped with redundant propulsion control. In the event of a specific thruster or rudder failing completely or experiencing partial malfunction, the lost control effect can be redistributed by other thrusters or rudders based on predetermined criteria. This redistribution enables the fault-tolerant control of the AUV propulsion system.

Based on the above analysis, during the operation of an AUV, the forces and moments at a specific moment can be represented using the state vector τ, where $\tau =\left[\tau_{x}\;\tau_{y}\;\tau_{z}\right]$ is the thrust force, and $\tau =\left[\tau_{m}\;\tau_{n}\right]$ is the moment generated by the control signal provided by the entire propulsion system. For the AUV control system, each state corresponds to a specific motion state, with forces and moments generated by the control signal *u*. In the context of AUV fault-tolerant control, the goal is to reconfigure a new set of thruster control signals based on the arrangement of the thrusters in the event of actuator faults. This reconfiguration ensures that the thruster can still provide sufficient force and moments to maintain control and stability of the AUV [28].

For the propulsion system arrangement described above, the force and moments formula is as follows:

(26) $$\tau =\left[\begin{array}{c} \tau_{x}\\ \tau_{y}\\ \tau_{z}\\ \tau_{m}\\ \tau_{n} \end{array}\right]=\left[\begin{array}{cccc} l\cos\alpha & \cos\alpha & \cos\beta & \cos\beta \\ \sin\alpha & \cos\alpha & 0 & 0\\ 0 & 0 & \cos\beta & \sin\beta \\ A & A & A & A\\ B & B & B & B \end{array}\right]\left[\begin{array}{c} u_{1}\\ u_{2}\\ u_{3}\\ u_{4} \end{array}\right]=B\cdot u$$

where $A=\frac{b}{2}\sin\alpha +\frac{a}{2}\cos\alpha ,\;B=\frac{b}{2}\cos\beta +\frac{a}{2}\sin\beta$.

In order to more intuitively reflect the utilization of thrust from each thruster and to prevent potential thrust saturation output when the multiple desired control quantities are simultaneously output, a detailed description of the normalization process for the output resultant thrust of the propulsion system is necessary. Assume the maximum control vector provided by the thruster can be represented as follows:

(27) $$\left\{\begin{array}{c} \tau_{xH}=2u_{m}\cos\alpha +2u_{m}\cos\beta =4u_{m}\cos\alpha =4u_{m}\cos\beta \\ \tau_{yH}=u_{m}\sin\alpha +u_{m}\cos\alpha \\ \tau_{zH}=u_{m}\sin\beta +u_{m}\cos\beta \\ \tau_{mH}=4A\\ \tau_{nH}=4B \end{array}\right.$$

By performing a conversion of the above equation, one can obtain the following:

(28) $$\left\{\begin{array}{c} {\bar{\tau }}_{x}=\frac{\tau_{x}}{u_{m}}=\frac{1}{4\cos\alpha }=\frac{1}{4}\cos\beta \\ {\bar{\tau }}_{y}=\frac{\tau_{y}}{u_{m}}=\frac{1}{2\sin\alpha }\\ {\bar{\tau }}_{z}=\frac{\tau_{z}}{u_{m}}=\frac{1}{2\sin\beta }\\ {\bar{\tau }}_{m}=\frac{\tau_{m}}{u_{m}}=\frac{1}{2}A\\ {\bar{\tau }}_{n}=\frac{\tau_{n}}{u_{m}}=\frac{1}{2B} \end{array}\right.$$

The final expression for the normalized state vector can be obtained as follows:

(29) $$\bar{\tau }=\left[\begin{array}{c} {\bar{\tau }}_{x}\\ {\bar{\tau }}_{y}\\ {\bar{\tau }}_{z}\\ {\bar{\tau }}_{m}\\ {\bar{\tau }}_{n} \end{array}\right]=\left[\begin{array}{cccc} l\frac{1}{4} & \frac{1}{4} & \frac{1}{4} & \frac{1}{4}\\ \frac{1}{2} & \frac{1}{2} & 0 & 0\\ 0 & 0 & \frac{1}{2} & \frac{1}{2}\\ \frac{1}{4} & \frac{1}{4} & \frac{1}{4} & \frac{1}{4}\\ \frac{1}{4} & \frac{1}{4} & \frac{1}{4} & \frac{1}{4} \end{array}\right]\left[\begin{array}{c} {\bar{u}}_{1}\\ {\bar{u}}_{2}\\ {\bar{u}}_{3}\\ {\bar{u}}_{4} \end{array}\right]=\bar{B}\bar{u}$$

For Equation (29), when the thrusters are operating normally, a certain thruster control voltage *u* will generate a corresponding rotational speed n, and then obtain the corresponding thrust and torque. When a fault occurs in the propulsion system, for the same control voltage, the output rotational speed n will decrease due to the presence of the fault, resulting in an inability to obtain the corresponding thrust and torque.

At this point, the thruster speed n can more accurately reflect the thrust and torque of the thruster during actual operation. In general, the relationship between the thrust and torque of a thruster and its rotational speed is a complex, non-linear one. However, for AUVs, this can often be approximated as a linear relationship. Therefore, Equation (31) can be used to calculate the actual force state of the AUV and compare it with the desired force state to evaluate the fault-tolerant control effectiveness.

(30) $$\bar{\tau }=\left[\begin{array}{c} {\bar{\tau }}_{x}\\ {\bar{\tau }}_{y}\\ {\bar{\tau }}_{z}\\ {\bar{\tau }}_{m}\\ {\bar{\tau }}_{n} \end{array}\right]=\left[\begin{array}{cccc} l\frac{1}{4} & \frac{1}{4} & \frac{1}{4} & \frac{1}{4}\\ \frac{1}{2} & \frac{1}{2} & 0 & 0\\ 0 & 0 & \frac{1}{2} & \frac{1}{2}\\ \frac{1}{4} & \frac{1}{4} & \frac{1}{4} & \frac{1}{4}\\ \frac{1}{4} & \frac{1}{4} & \frac{1}{4} & \frac{1}{4} \end{array}\right]\left[\begin{array}{c} {\bar{n}}_{1}\\ {\bar{n}}_{2}\\ {\bar{n}}_{3}\\ {\bar{n}}_{4} \end{array}\right]=\bar{B}\bar{n}$$

### 3.2. Pseudo-Inverse-Based Fault-Tolerant Control

The redundant configuration of the propulsion system in Section 2.2 allows for the fault-tolerant control of the thrusters. By utilizing the thruster priority matrix W, the control priority of each thruster can be expressed as follows:

(31) $$W=\left[\begin{array}{cccc} w_{1} & 0 & 0 & 0\\ 0 & w_{2} & 0 & 0\\ 0 & 0 & w_{3} & 0\\ 0 & 0 & 0 & w_{4} \end{array}\right]$$

In fault diagnosis and the fault-tolerant control of AUVs, the thruster priority matrix W is closely related to the thruster faults. When none of the structures in the propulsion system have failed, the priority of each thruster signal *u* is equal, i.e., $w_{1}=w_{2}=w_{3}=w_{4}=1$. In the event of a thruster fault, $f_{i}$ is considered as a fault factor. If the *i*-th thruster experiences complete failure, $f_{i}=0$. If the corresponding thruster experiences partial failure, then $0<f_{i}<1$.

Furthermore, the priority coefficients of partially failed thrusters can be represented as follows:

(32) $$w_{i}=e^{\frac{1}{f}-1}$$

If the thruster fails completely, $w_{i}=e^{+\infty }=+\infty$, then

(33) $$f_{i}=n_{i}\left(1-s_{i}\right)$$

where $n_{i}$ is the normalized thruster speed; $s_{i}$ is the congestion parameter. $0<s_{i}<1$ depends on the severity of the fault. If the thruster completely fails, $s_{i}=0$. For partial thruster faults, such as $s_{i}=0.5$, the output operating range of the thruster is limited to 50.

In this scenario, the fault-tolerant ability of the AUV system is analyzed in the event that a specific thruster encounters a time-varying fault. The affected thruster will experience a partial loss of driving function. The goal of the control system is to develop a new set of control signals to replace the original four thruster control signals that included the fault signal included in the fault occurrence. This ensures that the thruster can continue to generate the required thrust and torque even in the event of a fault, maintaining the AUV’s original motion states.

Assuming that the control signals of the thruster can meet the specified control quantity, the pseudoinverse allocation solution can be obtained using the Lagrange multiplier algorithm [29]. The Lagrangian function is defined as follows:

(34) $$L\left(u,\lambda \right)=\frac{1}{2}u^{T}Wu+\lambda \left(\tau -Bu\right)$$

where λ is a Lagrange multiplier. By taking the derivative of it, one can obtain:

(35) $$\frac{\partial L}{\partial u}Wu-B^{T}\lambda =0$$

Therefore, we can obtain:

(36) $$\tau =Bu=BW^{-1}B^{T}\lambda$$

Finally, the solution for pseudoinverse control allocation is obtained as follows:

(37) $$T={\bar{B}}^{+}u=\left[W^{-1}{\bar{B}}^{T}\left(\bar{B}W^{-1}{\bar{B}}^{T}\right)\right]u$$

where ${\bar{B}}^{+}$ is the pseudoinverse weight matrix of the thruster configuration matrix $\bar{B}$.

## 4. Fault-Tolerant Control of Actuators under Control Constraints

### 4.1. Description of Control Constraints

Section 3 studies the thrust allocation control using the pseudo-inverse method to ensure the normal operation of the AUV under ideal conditions. However, in practical operations, there are constraints on the input controls that drive the motion of the AUV. When the control inputs exceed the constraint limits, the pseudo-inverse method cannot automatically adjust the remaining control inputs to compensate for the insufficient control force caused by the constraints, leading to the actual control force failing to achieve the desired control effect.

**Figure 4:**
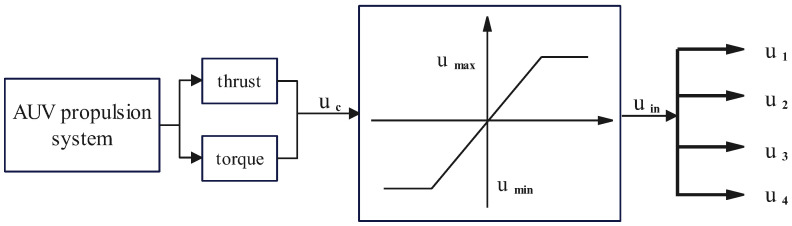
Schematic diagram of input saturation characteristics.

Due to the influence of its own dynamics and water flow, the propulsion system of an AUV has limitations and saturation nonlinearity in the forces and torques. Figure 4 illustrates the typical input saturation characteristics of the propulsion system.

The mathematical expression is described as follows:

(38) $$u_{in}=\left\{\begin{array}{c} u_{\max}\;\;u_{c}>u_{\max}\\ ku_{c}\;\;u_{\min}<u_{c}<u_{\max}\\ u_{\min}\;\;u_{c}<u_{\min} \end{array}\right.$$

When implementing fault-tolerant control in the presence of actuator faults, it is crucial to consider saturation constraints. In practical design, if the required control inputs exceed the range that the propulsion system can provide, the controller will continuously accumulate errors, resulting in system instability and potentially causing damage to the actuators.

Suppose that the insufficient control force and torque can be redistributed through the reallocation of the unsaturated control signals. The deviation matrix of the actuator control signal is denoted as *M*. If the maximum and minimum control input vectors are $T_{\max},T_{\min}$, the feasible range of the actuator control signal during reallocation is determined as follows:

(39) $$\left\{\begin{array}{c} M\leq T_{\max}-T\\ M\geq T-T_{\min} \end{array}\right.$$

and $B\left(T+M\right)=u$.

According to the properties of matrix multiplication, one can obtain:

(40) $$\left\{\begin{array}{c} BM=u-BT\\ BM=O_{m\times 1} \end{array}\right.$$

To ensure that the L2-norm of the control signals of the redistributed propulsion system is minimized, it is necessary to ensure that the L2-norm of the control signals obtained from the two control allocations is minimized.

According to the norm properties, one can obtain:

(41) ∥T+M∥≤∥T∥+∥M∥

Furthermore, the problem is transformed into solving $\min∥M_{dev}∥$. Finally, the aforementioned problem can be formulated in the following standard form of quadratic programming:

(42) $$\begin{array}{ll} \min & J=M^{T}HM+CM\\ s.t. & A_{i}M+B_{i}=0,\;i\in E\\ & A_{i}M+B_{i}\geq 0,\;i\in I \end{array}$$

where *H* is the Hessian matrix; *C* is the coefficient matrix of the linear term for the variable *M*; $A_{i},\;B_{i}$ are the coefficient matrices of the linear terms and constant terms in the constraint functions for the variable *M*, respectively.

### 4.2. Nonlinear Quadratic Programming

The nonlinear quadratic programming algorithm is efficient in solving nonlinear programming problems. Compared to other algorithms, its most prominent advantages include its good convergence, high computational efficiency, and strong boundary search capabilities. It has been widely used in many fields. All non-linear programming models are generally composed of an optimization objective function and corresponding constraints. For optimization problems with inequality constraints, this paper uses the active set method to solve quadratic programming, shown as follows.

Assuming $\bar{M}$ is a feasible point for Equation (44), its active set can be defined as the set of constraints for which the equality holds, that is:

(43) $$A\left(\bar{M}\right)=\left\{i\in \left(E\cup I\right):A_{i}\bar{M}=B_{i}\right\}$$

For quadratic programming problems with inequality constraints, the Lagrangian function can be described as follows:

(44) $$L\left(M,\lambda \right)=M^{T}HM+CM-\sum_{i\in \left(E\cup I\right)}\left[\lambda_{i}\left(A_{i}\bar{M}-B_{i}=0\right)\right]$$

Furthermore, the optimality condition (K-T) for quadratic programming can be described as follows:

(45) $$H\bar{M}+C-\sum_{i\in A\left(\bar{M}\right)}\lambda_{i}A_{i}=0$$

where

(46) $$A_{i}\bar{M}=B_{i},i\in A\left(\bar{M}\right)\;A_{i}\bar{M}\geq B_{i},i\in I\cup A\left(\bar{M}\right)\;\lambda_{i}\geq 0,i\in I\cap A\left(\bar{M}\right)$$

and $\bar{M}$ is also referred to as the K-T point of Equation (46).

The active set method is essentially an iterative algorithm that uses the feasible point of Equation (46) as the starting point in each iteration and defines the search direction with the set of active constraints, then determines the search step size through linear search.

Let $M_{k}$ be the feasible solution at the k-th step, with its active set denoted as $S_{k}=E\cup I\left(M_{k}\right)$; consider the following optimization problem:

(47) $$\min\left(M^{T}HM+C^{T}M\right)\;s.t.\;\;A_{i}M=B_{i},i\in S_{k}$$

Denote the step length $p=M-M_{k}$ and substituting it into Equation (47) while removing the constant term yields the equivalent optimization problem, as follows:

(48) $$\begin{array}{l} \min\left(p^{T}Hp+p_{k}^{T}p\right)\\ s.t.\;\;A_{i}p=B_{i},i\in S_{k} \end{array}$$

where $p_{k}=p+HM_{k}$. Find the optimal solution $p_{k}$ of quadratic programming under equality constraints and divide it into the following three cases:

(1) $p_{k}\neq 0$, while $M_{k}+p_{k}$ is a feasible point of Equation (47). Take a new iteration point $M_{k+1}=M_{k}+p_{k}$ and then calculate the corresponding optimization problem.

(2) $M_{k}+p_{k}$ is a feasible point of Equation (47). Denote $M_{k+1}=M_{k}+\alpha_{k}p_{k}$, where the step size parameter $\alpha_{k}\in \left(0,1\right]$ can be represented in detail as follows to ensure that $M_{k+1}$ in (2) is a feasible point.

(49) $$\alpha_{k}=\min\left\{1,\min\frac{B_{i}-B_{i}M_{k}}{A_{i}p_{k}}\right\}$$

(3) $p_{k}=0$, $M_{k}$ is the optimal solution of the optimization problem (48). Determining $M_{k}$ in (3) is the K-T point of the original problem. If all Lagrange multipliers of the original problem are non-negative, it can be concluded that this point is the optimal solution of the quadratic programming problem (47) based on the convex optimization theory.

Quadratic programming can be used to optimize and solve the problems of minimizing tracking errors and control inputs. When the propulsion system fails, quadratic programming can dynamically update actuator matrices based on information, achieving re-allocation under control constraints.

## 5. Experimental Verification and Analysis

### 5.1. Simulation Settings

In this section, simulations are conducted for the actuator fault-tolerant control of an AUV with known fault types. The faults are set as follows: (1) Thruster fault: at 150 s, one of the thrusters experiences a thrust degradation to 0, while the parameters of the other thrusters remain unchanged; (2) Rudder servo fault: at 150 s, one of the servos fails, with its torque degrading to 0, rendering it unable to deflect and provide the designed torque, while the parameters of the other servos remain unchanged. It should be noted that the two types of fault conditions mentioned in this article will not occur simultaneously and are not related to each other.

### 5.2. Simulation Results and Analysis

(1) Fuzzy controller input signals

**Figure 5:**
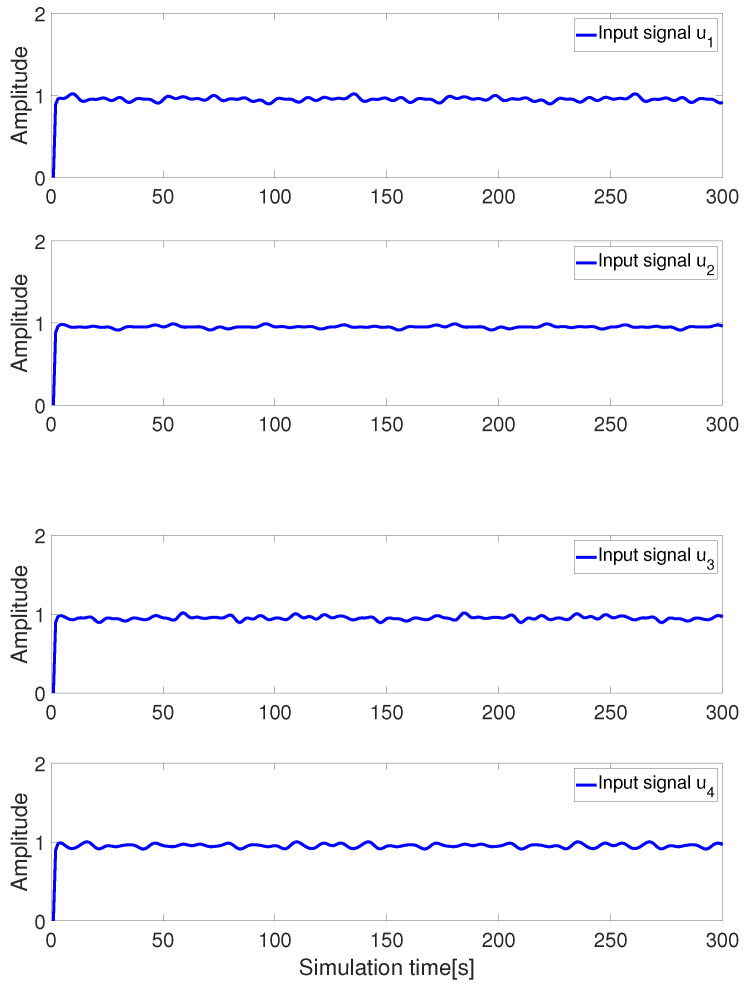
Input signal for fuzzy controller.

Figure 5 illustrates the control input signals generated by the T-S fuzzy controller for the actuator of the AUV, which are provided by the thrusters and the rudder servo. From the figure, it can be observed that the input control signals generated by the fuzzy controller are stable, indicating that the actuators of the AUV can provide a stable thrust and torque to the AUV.

(2) Pseudo-inverse-based fault-tolerant control (without control constraints)

**Figure 6:**
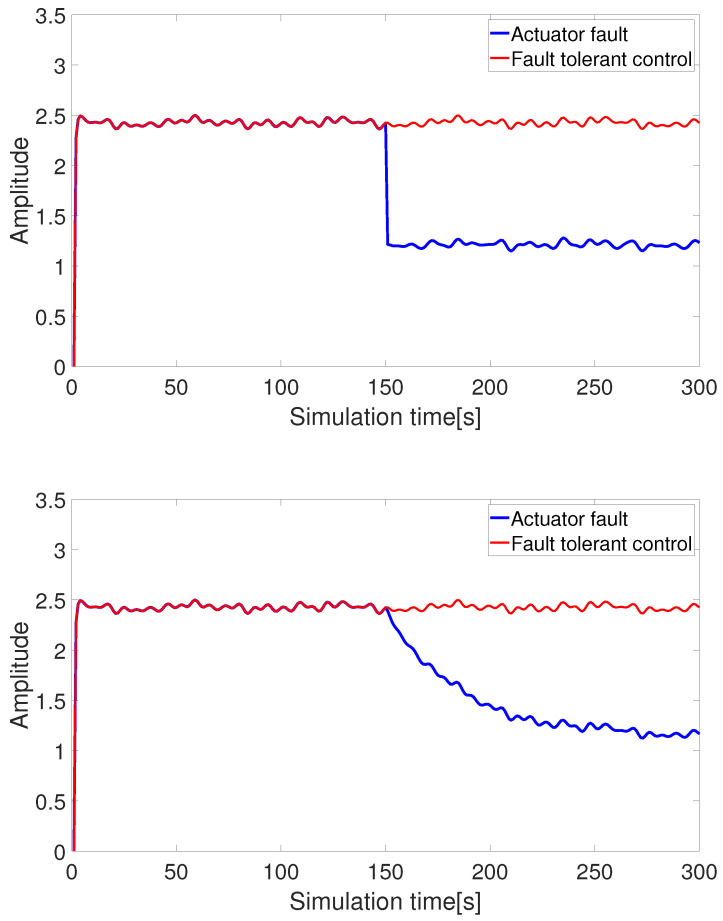
Fault-tolerant control under thrust loss (abrupt fault + slowly-varying fault).

**Figure 7:**
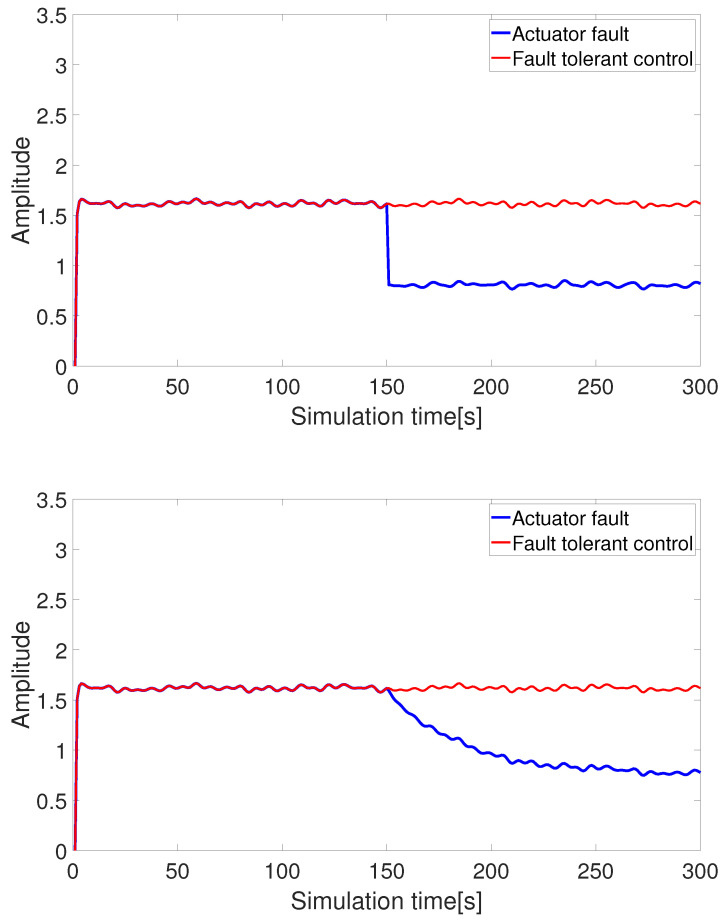
Fault-tolerant control under moment loss (abrupt fault + slowly-varying fault).

Figure 6 and Figure 7 show the pseudo-inverse fault-tolerant control based on thrust allocation. It can be observed that when the control inputs of the AUV actuator degrade due to faults, the output states change accordingly. By employing the pseudo-inverse method for the thrust allocation of faulty thrusters and rudders, the lost thrust and torque can be effectively compensated, enabling the AUV to maintain a certain level of system dynamic performance even in the presence of faults.

(3) Quadratic programming under control constraints

**Figure 8:**
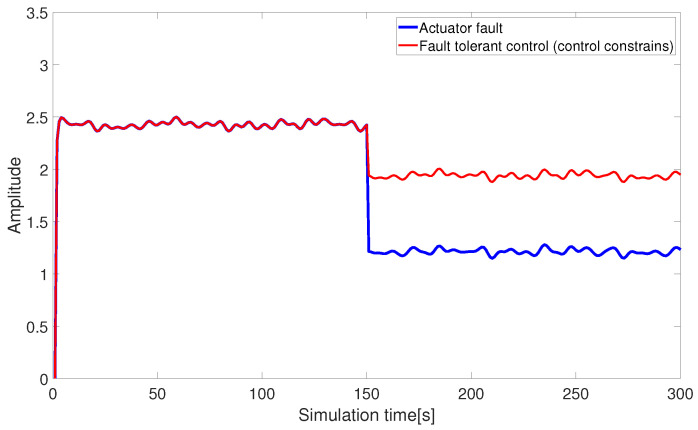
Fault-tolerant control under control constraints (abrupt fault).

**Figure 9:**
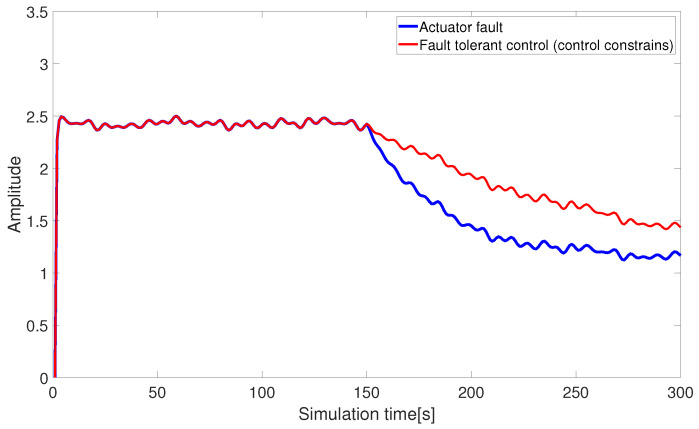
Fault-tolerant control under control constraints (slowly varying fault).

Figure 8 and Figure 9 illustrate the thrust allocation results based on pseudo-inverse control under control constraints. When the control input fails to provide the ideal signal due to various factors, the pre-designed control signals exceed the constraint range. The original fault-tolerant thrust allocation method is unable to fully compensate for the missing effectiveness due to faults, resulting in the originally designed fault-tolerant control scheme failing to meet the expected effectiveness, which leads to significant deficiencies in practical applications.

**Figure 10:**
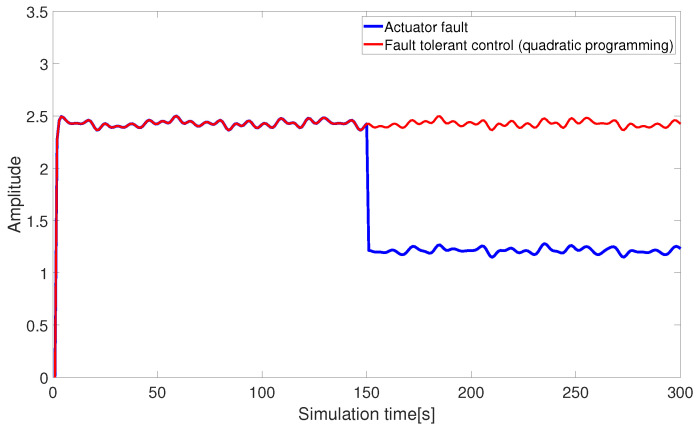
Fault-tolerant control under quadratic programming (abrupt fault).

**Figure 11:**
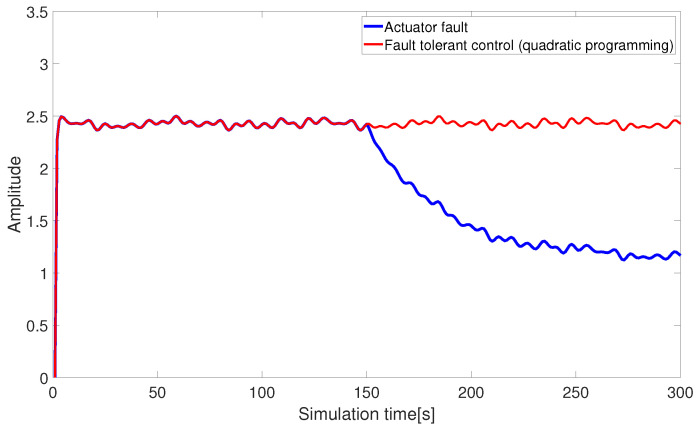
Fault-tolerant control under quadratic programming (slowly-varying fault).

Figure 10 and Figure 11 present the fault-tolerant control results of the AUV actuators, combined with pseudo-inverse and nonlinear quadratic programming. The quadratic programming algorithm compensates for the missing control input within control constraints, allowing the input thrust of the AUV actuator to return to normal and achieving the expected fault-tolerant effect.

**Figure 12:**
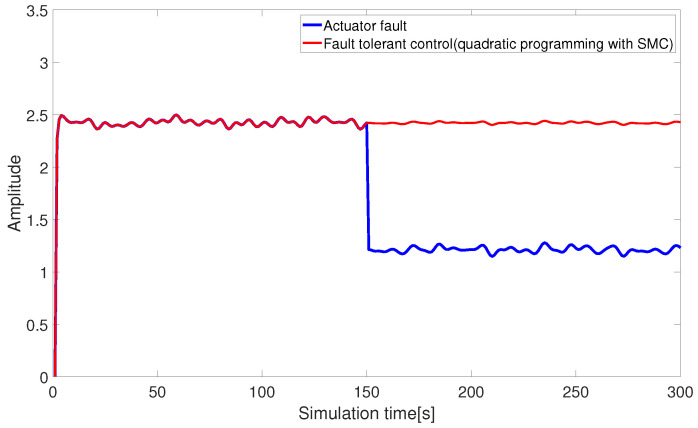
Fault-tolerant control under quadratic programming and SMC (abrupt fault).

**Figure 13:**
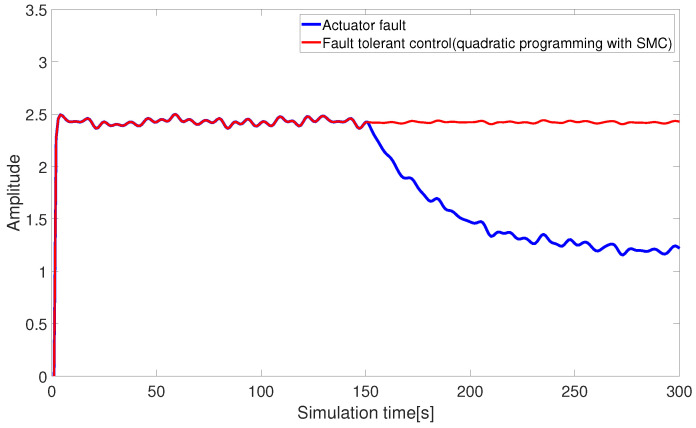
Fault-tolerant control under quadratic programming and SMC (slowly-varying fault).

In general, the T-S fuzzy logic and pseudo-inverse quadratic programming-based fault-tolerant scheme in this paper can resolve the issue of AUV actuator faults under control constraints. However, in terms of fault-tolerant controller design, the proposed solution in this paper does not consider the requirement for robustness. Inspired by Ref. [30], sliding mode control (SMC) exhibits strong robustness to external disturbances and parameter uncertainties, with a good response and tracking performance, making it suitable for scenarios with high demands on system dynamic performance. Therefore, as shown in Figure 12 and Figure 13, the SMC approach can be taken into consideration in the design of fault-tolerant controllers for both abrupt faults and slowly varying faults based on the previous quadratic programming. Furthermore, based on the comparisons in Figure 10, Figure 11, Figure 12 and Figure 13 (as indicated by the solid red line), the fault-tolerant control obtained under quadratic programming and SMC design can obtain advanced robustness without sacrificing the nominal control performance of AUVs.

## 6. Conclusions

In this paper, we propose a fault-tolerant control method for AUV actuators based on T-S fuzzy logic and pseudo-inverse quadratic programming, taking into account control constraints in practical scenarios. Additionally, the thrust allocation method, employing pseudo-inverse quadratic programming, has demonstrated effective fault-tolerant control for AUV actuators. The simulation results indicate that the proposed fault-tolerant control method with control constraints can effectively compensate for missing control input signals, ensuring the normal operation of AUVs in the event of actuator faults. Our future work will focus on efficient fault diagnosis methods [31,32,33] to obtain comprehensive fault information for AUVs to further improve fault tolerance, and the data from real-world experiments (not only the numerical simulations) will be used to demonstrate the controller’s performance in practical scenarios.

## Figures and Tables

**Table 1:** Coordinate system-related parameter definition for AUV [26].

Symbols	Descriptions	Units
X	The force on x axis	N
Y	The force on y axis	N
Z	The force on z axis	N
K	The moment on x axis	N·m
M	The moment on y axis	N·m
N	The moment on z axis	N·m
p, q, r	Roll, pitch and heading angular velocity	rad/s
u, v, w	The velocity on x, y and z axes	m/s
φ, θ, ψ	Roll angle, pitch angle, heading angle	rad

## Data Availability

The data presented in this study are available on request from the corresponding author.

## Abbreviations

The following abbreviations are used in this manuscript:

AUV	Autonomous Underwater Vehicle
T-S	Takagi and Sugeno
FTESO	Finite-Time Extended State Observer
